# Small-Pox Specifics—Is Vaccination a Failure

**Published:** 1887-10-15

**Authors:** 


### Small-Pox Specifics—Is Vaccination a Failure?

By Mr. William Tebb.

In the recent Vaccination Debate in Parliament, the President of the Local Government Board, Mr. Ritchie, according to Hansard's Report, said : "Nurses of London small-pox hospitals never catch the disease ; and there has never been a single case amongst them." This statement was astounding to members of Parliament and others interested in this important question who have devoted years to its investigation, and know the true facts. In March 1885, Mr. G. Russell, the then Secretary to the Local Government Board, reported in the House, in reply to a question, that four revaccinated nurses had been smitten with small-pox at Stockwell since 1876, four at Fulham, and one at Deptford, and Mr. Ritchie could, had he taken the trouble to inquire, have discovered from the medical evidence before the Vaccination Committee of 1870, that many of the nurses are selected because they have already had small-pox. But it is obvious that facts showing the failure of vaccination and revaccination are apt to meet with dignified disapproval, even when, as in these cases, they have been officially admitted in Parliament by a high official of the department. Why should it be supposed that revaccination confers immunity to small-pox hospital nurses, when the same revaccination has been proved by Mr. A. R. Wallace ("Forty-five Years' Registration Statistics") to have conspicuously failed amongst the revaccinated troops? On the 23rd March 1885, the Marquis of Hartington, in reply to a question submitted by Mr. C. H. Hopwood, admitted that since the arrival of the troops in Egypt in 1882, eighty-one cases of small-pox had occurred, of which seven had proved fatal; fifty-one of these were known to have been duly revaccinated, and the rest believed to have been so. Indeed, the custom of the service is to carefully revaccinate all recruits immediately on their acceptance, so that here are eighty-one revaccinated failures.

In the presence of such glaring inaccuracies by chiefs of public departments, it is surely time that members of Parliament should pause before blindly adopting the unsupported statements of medical specialists, when advanced to impose, by means of ignominious punishments, the disputed and ever-changing dogmas of a pre-scientific age upon English freemen.

---

By John C. McVail, M.D.,

Sanit. Sci. Certif. Camb.

In the absence of Mr. Shirley Murphy, who is abroad, we sent Mr. Tebb's letter to Dr. McVail, of Kilmarnock, who is entitled to be considered an authority on this question. Truth often lies at the bottom of a well, and by full and impartial discussion the public can alone arrive at the real bearings and true facts of this question. How easily a man who is a partisan may deceive himself, is once more shown by the following statement, which Dr. McVail has sent us in reply to the points raised by Mr. Tebb :—

Mr. Ritchie was quite correct in his statement that revaccinated "nurses of London small-pox hospitals never catch the disease ; and there has never been a single case amongst them." Mr. Russell in Parliament seems to have been founding on Dr. Sweeting's "Memorandum of Vaccination," issued from Fulham Hospital in 1882, and it referred, not to nurses alone, but to porters, kitchen and laundry staff, etc., among whom revaccination seems to have been not so rigidly enforced nor so carefully recorded as among those in actual attendance on the sick. For example, as to the four cases occurring at Fulham (none of which were in nurses), Mr. Sweeting has recently informed me that "in two, the operation (of revaccination) was unsuccessful, and was not repeated," and that as to the failure or success of the other two, no record has been left by his predecessor.

Quite recently the Vaccination Committee of the Epidemiological Society appears to have gone over the evidence afresh, confining its attention to those "in personal attendance on cases of small-pox." In the Metropolitan Board Hospitals, of 734 nurses and attendants, 79 had had previous small-pox, and escaped subsequent infection ; 645 had been revaccinated just before entering on their duties, and not one of these took small-pox ; ten had escaped revaccination, and every one of them contracted small-pox. These ten exceptions are very useful in proving the rule, and showing that it is not to any masculine character, nor to "gradual exposure," that nurses owe their immunity, but to revaccination alone.

The above figures show the amount of truth in the contention "that many of the nurses are selected because they have already had small-pox." But what does Mr. Tebb mean by this contention? Does he believe that one attack of small-pox protects against a second? Let him give a plain Yes, or No. And if he does not believe it, then why does he thus appeal to what he must hold to be a popular delusion? My reason for asking this question is, that an important plank in the anti-vaccination platform consists in the assertion that vaccine lymph is simply small-pox virus unmodified as to its infective power. Now it is obvious that if they are the same, they must have the same power. If Mr. Tebb believes this, does he also grant that vaccine prevents small-pox?—if not, what does he mean by suggesting that one attack of small-pox prevents a second? It is not true that Dr. A. R. Wallace has proved "revaccination to have conspicuously failed amongst the revaccinated troops." It is Dr. Wallace who, in spite of his acknowledged ability, has "conspicuously failed" to prove this. He says that the small-pox rate in England and Wales in 1860-82 inclusive, was, among adult males, 176 per million, while in the Army it was barely 83 per million. True, in the Navy it was 157 per million; and while Dr. Wallace ingeniously speculates as to the possibility of this difference between the Services being due to different hygienic surroundings, he is apparently ignorant of the fact that the explanation is much simpler—namely, that in the Navy revaccination was not compulsory till 1871!

Moreover, in the Forces, as among the nurses, we find the exceptions proving the rule. For in his report for 1884, Dr. George Buchanan says, referring to a return obtained by a prominent anti-vaccinator, Mr. Burt, M.P.:—

"As regards small-pox in the Navy, the forty-three deaths shown by Mr. Burt's return to have occurred in that Service in the eleven years following the order of 1871, are found, on reference to the reports of that de-department, to be made up of thirteen persons who were vaccinated once, and once only; of twelve persons (including eleven Kroomen) who had never been vaccinated at all; of twelve persons (including two foreigners) about whom no information was to be had; and of six persons who had presumably been successfully vaccinated and re-vaccinated. These were the small-pox deaths occurring during eleven years, on a mean strength of nearly 60,000 men."

Where now are the "glaring inaccuracies by chiefs of public departments"? Are we not rather in presence of glaring inaccuracies by chiefs of the Anti-Vaccination Society?

